# Population-agnostic real-time molecular mismatch estimation using rSSO-defined HLA allele strings: minimal impact of allelic discordance with NGS

**DOI:** 10.3389/fimmu.2026.1781594

**Published:** 2026-03-11

**Authors:** Raja Rajalingam, Pawan Kumar Raghav, Neelam Sharma, Gilberto Da Gente, Denice Kong

**Affiliations:** Immunogenetics and Transplantation Laboratory, Department of Surgery, University of California, San Francisco, San Francisco, CA, United States

**Keywords:** eplet mismatch, high-resolution HLA typing, HLA imputation, HLA matching, HLA typing, molecular mismatch, next-generation sequencing (NGS), organ transplantation

## Abstract

**Background:**

Molecular HLA mismatch at the epitope level is strongly associated with donor-specific antibody formation, antibody-mediated rejection, and graft failure after transplantation. Accurate molecular mismatch assessment requires high-resolution HLA typing; however, in clinical practice—particularly in deceased donor transplantation—typing is often performed at low or intermediate resolution. Haplotype-based imputation methods have been developed to infer high-resolution alleles but show variable accuracy, particularly in underrepresented populations. We evaluated whether the first allele listed in the reverse sequence-specific oligonucleotide (rSSO)–derived National Marrow Donor Program (NMDP) multiple allele code (MAC) string can serve as a practical surrogate for high-resolution typing in molecular mismatch assessment.

**Methods:**

We analyzed 4,738 individuals who underwent dual HLA typing by rSSO and next-generation sequencing (NGS) across 11 classical HLA loci. Concordance between the first allele of the rSSO MAC string and the corresponding two-field NGS allele was assessed. For discordant allele pairs, molecular disparity was quantified by calculating eplet mismatches using the HLA Eplet Mismatch Calculator.

**Results:**

Allele-level concordance exceeded 94% for 9 of 11 loci, including HLA-A (97.9%), -B (98.5%), -C (95.2%), -DRB1 (96.8%), -DRB3 (94.5%), -DRB5 (99.6%), -DQB1 (98.3%), -DPB1 (96.8%), and -DPA1 (98.7%). Lower concordance was observed for HLA-DQA1 (86.7%) and -DRB4 (72.0%). Most discordant NGS alleles, except for the DPB1 locus, were present in the rSSO MAC string. Among discordant allele pairs, 30.4% had zero eplet mismatches and 77.6% had two or fewer mismatched eplets. Allele pairs with more than two mismatched eplets accounted for less than 0.3% of all comparisons across loci. No significant differences in concordance were observed across racial or ethnic groups.

**Conclusion:**

The first allele listed in the rSSO-derived MAC string provides a reliable, population-agnostic surrogate for high-resolution HLA typing in molecular mismatch assessment. Although allele-level discordance occurs, it rarely results in clinically meaningful increases in eplet mismatch burden. This laboratory-based approach enables real-time molecular mismatch estimation in time-sensitive settings and facilitates retrospective analysis of cohorts lacking sequencing-based HLA data, supporting broader integration of molecular mismatch into precision transplantation practice.

## Introduction

Organ transplantation remains the definitive therapy for patients with end-stage organ failure; however, long-term graft survival continues to be limited by alloimmune injury (1–4). Genetic disparity between donor and recipient within the human leukocyte antigen (HLA) system is a major determinant of this risk and underlies the development of donor-specific antibodies (DSA), antibody-mediated rejection (AMR), and graft failure (5). Traditional antigen-level HLA matching offers only a coarse estimate of compatibility, as it fails to capture the molecular determinants of alloimmunity—specifically, the amino acid–level epitopes recognized by B and T cells.

To overcome this limitation, molecular mismatch algorithms such as HLAMatchmaker (6), PIRCHE-II (7, 8), and EMMA (9) were developed to quantify donor–recipient differences at the epitope or peptide level. A growing body of evidence demonstrates that higher molecular mismatch burdens are strongly associated with *de novo* DSA formation, AMR, and inferior graft survival across kidney, heart, and lung transplantation cohorts (10–14). Consequently, molecular mismatch has emerged as a promising biomarker for immunologic risk stratification and individualized immunosuppression.

Accurate assessment of molecular mismatch requires high-resolution (two-field or higher) HLA typing, as even a single amino acid difference can alter epitope structure and immunogenicity. However, in clinical practice, particularly in deceased donor transplantation, HLA typing is typically performed at low or intermediate resolution due to time constraints. As a result, high-resolution data are often unavailable both prospectively during organ allocation and retrospectively in large clinical datasets.

Several imputation strategies have been developed to address this gap, inferring high-resolution HLA alleles from low-resolution typing based on population haplotype frequencies and linkage disequilibrium. The most widely used tool, NMDP HaploStats (https://www.haplostats.org), provides allele-level inference across multiple loci but achieves error-free predictions in only approximately one-third of cases, with substantially reduced accuracy in non-Caucasian populations (~29%) compared with Caucasians (~45%) (15). Other approaches, such as the HLAMatchmaker converter (6), perform even less reliably, correctly imputing fewer than half of cases in some studies (15), and are further constrained by their reliance on a limited set of reference populations. These inaccuracies introduce uncertainty into molecular mismatch estimates, complicating their clinical interpretation.

Most validation studies of imputation tools have focused on low-resolution (one-field) typing. Whether intermediate-resolution typing, commonly generated by reverse sequence-specific oligonucleotide (rSSO) assays, can serve as a more reliable proxy for high-resolution alleles has not been systematically evaluated. In this study, we leveraged a large cohort of 4,738 individuals who underwent dual HLA typing using both rSSO and next-generation sequencing (NGS) across 11 classical HLA genes. We assessed concordance between the first allele listed in the NMDP multiple allele code (MAC) generated by rSSO typing and the corresponding NGS-resolved allele. We further quantified the molecular mismatch introduced by discordant calls using eplet analysis. Our objective was to determine whether the first allele of the rSSO MAC string can function as a practical surrogate for high-resolution typing in molecular mismatch assessment when sequencing data are unavailable. Because rSSO directly interrogates allelic variation in the laboratory, it avoids reliance on population reference panels and statistical inference based on linkage disequilibrium. This makes rSSO a population-agnostic alternative to haplotype-based imputation methods, whose performance may vary across populations that are underrepresented in reference datasets. If validated, this approach could bypass the need for ethnicity-specific imputation and computational inference, enabling real-time application in deceased donor allocation and retrospective mismatch analysis. Clarifying these capabilities and limitations is essential for advancing the integration of molecular mismatch into routine clinical practice and supporting precision medicine in transplantation.

## Materials and methods

### Study cohort and HLA typing

This retrospective study was conducted at the Immunogenetics and Transplantation Laboratory (ITL) at the University of California, San Francisco (UCSF), California, USA, and was approved by the UCSF Institutional Review Board (IRB #16-19103). HLA allele typing data were retrieved for 7905 samples that were typed using NGS between November 2017 and June 2023. Excluded from further analysis were 2843 samples for which rSSO typing was not performed, 274 samples lacking rSSO results for one or more HLA genes, and 50 samples for which NGS data were lacking for one or more HLA genes. The remaining 4,738 samples (9,476 alleles per gene) had complete HLA typing results for 11 HLA genes from both the rSSO and NGS methods. The cohort was racially and ethnically diverse. Ethnicity information was not available for 30% of individuals. Among those for whom information was available, white individuals comprised the largest group (34.3%), followed by Hispanic/Latino individuals (31.4%). Asian individuals accounted for 18.5%, African Americans for 11.9%, and individuals classified as other races or ethnicities represented 3.9%. DNA extraction and HLA typing by rSSO and NGS were performed as described previously (16).

### Concordance analysis

NGS typing results were reported as fully resolved alleles, whereas rSSO typing results were reported with the multiple allele code (MAC) established by the National Marrow Donor Program (NMDP) (https://www.transplanttoolbox.org/allan/codes). Each NMDP MAC code corresponds to a string of alleles consistent with the rSSO typing results. The NGS result, at two-field level resolution, was compared to the first allele of the rSSO determined allele string, and when the results were a match, it was counted as concordant. Results were categorized as both alleles concordant, one-allele discordant, or two-allele discordant. Discordant results were further analyzed to determine whether the NGS-typed allele was included among the remaining alleles in the rSSO allele string. For HLA-DRB3, -DRB4, and -DRB5, the absence of a gene was treated as an allele.

### Eplet mismatch assessment

For discordant allele pairs, molecular mismatches were quantified by calculating eplet mismatches using the HLA Eplet Mismatch Calculator (version 2025-11-07. IPD-IMGT/HLA: 3.62; https://epregistry.com.br/calculator). The HLA Eplet Mismatch Calculator was selected because it implements an outcome-validated, epitope-based framework derived from HLAMatchmaker that enables direct allele-level comparison across both class I and class II HLA loci (6). Eplet mismatches defined within this framework have been consistently associated with donor-specific antibody development, rejection, and graft outcomes across multiple transplant cohorts (17–20). In contrast, alternative tools address different immunologic mechanisms or apply distinct analytic approaches: PIRCHE-II estimates indirectly presented donor-derived peptides relevant to T-cell–mediated alloreactivity (7, 8), while EMMA applies physicochemical amino acid distance metrics (9) that are not directly aligned with established eplet mismatch thresholds. Accordingly, we used the antibody-verified eplet-based framework of the HLA Eplet Mismatch calculator to evaluate whether rSSO-derived allele assignments preserve clinically meaningful molecular mismatch relative to high-resolution NGS typing. Fully resolved two-field HLA alleles were used as input for both class I (HLA-A, -B, -C) and class II (HLA-DRB1, -DRB3/4/5, -DQA1, -DQB1, -DPA1, -DPB1) loci. The calculation was performed unidirectionally with the first allele of the rSSO MAC string treated as the donor allele and the NGS-typed allele as the recipient allele. The number of eplet mismatches was then determined for each discordant pair. Default calculator settings were applied. Null, novel, or questionable alleles were excluded from eplet mismatch calculations due to the absence of defined eplet structures.

## Results

In this study, we compared the HLA typing results obtained from high-resolution next-generation sequencing (NGS) with those from intermediate-resolution reverse Sequence-Specific Oligonucleotide (rSSO) typing. We used the first allele of the NMDP MAC string reported in the rSSO typing results as a proxy for the typing result and compared it with the allele determined by NGS to assess concordance. **Table 1** shows example outcomes from this analysis. Results were reported as both alleles concordant, one-allele discordant, or both alleles discordant. In the cases of discordance, it was further determined whether the discordant NGS-determined allele was found in the rSSO-reported allele string.

**Table 1:** Examples of rSSO typing results compared with NGS types and inferred concordance.

Examples	rSSO typing results		NGS types	Inference
	NMDP code	Allele string*		
1	A*11: SJFW	**11:01**/11:69N/11:86/11:97/11:99N/11:100/11:104/11:107/11:109N	11:01	Both alleles concordant
	A*24: RXWX	**24:02**/24:144/24:150/24:154/24:155N/24:163N/24:175/24:178/24:179/24:185N/24:186	24:02	
2	A*29: CXJSN	29:01/**29:02**/29:44/29:100/29:106/29:109/29:111/29:119/29:120/29:121/29:126Q/29:128/29:131/29:134/29:135/29:136/29:137/29:140/29:142	29:02	One allele discordant and included in string
	A*68: DFRKT	**68:01**/68:101/68:116/68:119/68:120N/68:152/68:167/68:189/68:202/68:203N/68:205N/68:207/68:208/68:210N/68:217/68:228/68:229/68:243/68:245N/68:248	68:01	
3	B*35: BPTKA	35:01/35:42/35:57/35:119/35:131/35:173/35:324/35:327/35:330/35:332/35:347/35:348/35:353/35:359/35:363/35:365/35:370/35:376/35:380/35:400/35:406/35:409	35:241	One allele discordant and excluded from string
	B*14: BMUJS	**14:02**/14:64/14:67	14:02	
4	A*11: AD	11:01/**11:04**	11:04	Both alleles discordant but included in strings
	A*34: CVZNW	34:01/**34:05**/34:18/34:23	34:05	
5	DRB1*04: AYGNB	04:01/04:151/04:171/04:190/04:192/04:216/04:233	04:05	Both alleles discordant and excluded from strings
	DRB1*13: AYHMF	13:01/13:105/13:112/13:117/13:166/13:173/13:186/13:190/13:200N/13:201/13:213/13:215/13:218/13:222/13:226/13:233/13:238	04:11	

*concordant alleles are indicated in bold lettering.

Although no gene showed 100% allele concordance, nine of the eleven loci showed greater than 94% concordance: HLA-A (97.9%), -B (98.5%), -C (95.2%), -DRB1 (96.8%), -DRB3 (94.5%), -DRB5 (99.6%), -DQB1 (98.3%), -DPB1 (96.8%) and -DPA1 (98.7%) (**Table 2**). Locus-specific discordant allele patterns and frequencies are provided in **Supplementary Tables 1**–**11**. HLA-DQA1 demonstrated lower concordance at 86.7%. Among discordant DQA1 alleles, 98% were represented within the NMDP MAC string. Of the remaining discordant alleles (21 total), nine were newly identified alleles (**Supplementary Table 9**) and were therefore not included in the database used to generate the NMDP MAC strings. No significant differences in allele concordance or discordance were observed across racial or ethnic groups.

**Table 2:** Allele-level concordance and discordance between rSSO and NGS typing across HLA loci.

HLA Loci (4738 Sample; 9476 alleles per locus)	Total alleles concordant	Both alleles concordant	One allele concordant and another allele discordant				Both alleles discordant*		
			Concordant alleles	Discordant alleles			Total	Both alleles included in string	Both alleles excluded from string
				Total	Included in string	Excluded from string			
	% (count)	% (count)	% (count)	% (count)	% (count)	% (count)	% (count)	% (count)	% (count)
A	97.9 (9281)	95.9 (9090)	2.0 (191)	2.0 (191)	88.5 (169)	11.5 (22)	0 (4)	100 (4)	0 (0)
B	98.5 (9334)	97.0 (9192)	1.5 (142)	1.5 (142)	97.2 (138)	2.8 (4)	0 (0)	0 (0)	0 (0)
C	95.2 (9020)	90.5 (8574)	4.7 (446)	4.7 (446)	97.5 (435)	2.5 (11)	0.1 (10)	100 (10)	0 (0)
DRB1	96.8 (9177)	94.0 (8904)	2.9 (273)	2.9 (273)	96.7 (264)	3.3 (9)	0.3 (26)	76.9 (20)	23.1 (6)
DRB3	94.5 (8954)	89.3 (8458)	5.2 (496)	5.2 (496)	99.4 (493)	0.6 (3)	0.3 (26)	53.8 (14)	46.2 (12)
DRB4	72.0 (6827)	58.6 (5550)	13.5 (1277)	13.5 (1277)	99.0 (1264)	1.0 (13)	14.5 (1372)	95.6 (1311)	4.4 (61)
DRB5	99.6 (9442)	99.4 (9416)	0.3 (26)	0.3 (26)	100 (26)	0 (0)	0.1 (8)	50.0 (4)	50.0 (4)
DQB1	98.3 (9318)	96.7 (9162)	1.6 (156)	1.6 (156)	92.3 (144)	7.7 (12)	0 (2)	0 (0)	100 (2)
DQA1	86.7 (8220)	75.5 (7158)	11.2 (1062)	11.2 (1062)	98.5 (1046)	1.5 (16)	2.0 (194)	97.4 (189)	2.6 (5)
DPB1	96.8 (9173)	94.0 (8906)	2.8 (267)	2.8 (267)	0.7 (2)	99.3 (265)	0.4 (36)	0 (0)	100.0 (36)
DPA1	98.7 (9350)	97.4 (9230)	1.3 (120)	1.3 (120)	95.8 (115)	4.2 (5)	0.1 (6)	66.7 (4)	33.3 (2)

*No samples were observed where one allele was included and the other excluded among both allele discordant pairs.

The HLA-DRB3, -DRB4, and -DRB5 genes are variably present depending on the underlying haplotype. For the purposes of this study, gene absence was treated as an allele to allow consistent concordance analysis. Allele concordance differed among the three loci: HLA-DRB5 showed the highest concordance (99.6%), followed by HLA-DRB3 (94.5%), while HLA-DRB4 demonstrated the lowest concordance (72.0%; **Table 2**). Among all loci analyzed, HLA-DRB4 had the highest proportion of discordant alleles (28%). Despite this, the majority of discordant DRB4 alleles (97.2%) were included within the rSSO-reported allele string (**Supplementary Table 6**). A single allele, HLA-DRB4*01:03, accounted for nearly all NGS typing results categorized as discordant but still included in the reported string, with only three exceptions. In cases where the DRB4 allele was not included in the rSSO-reported allele string, the predominant allele was DRB4*01:03N. This allele accounted for all but four such typing results, one of which represented a newly identified DRB4 allele (**Supplementary Table 6**).

**Figure 1:**
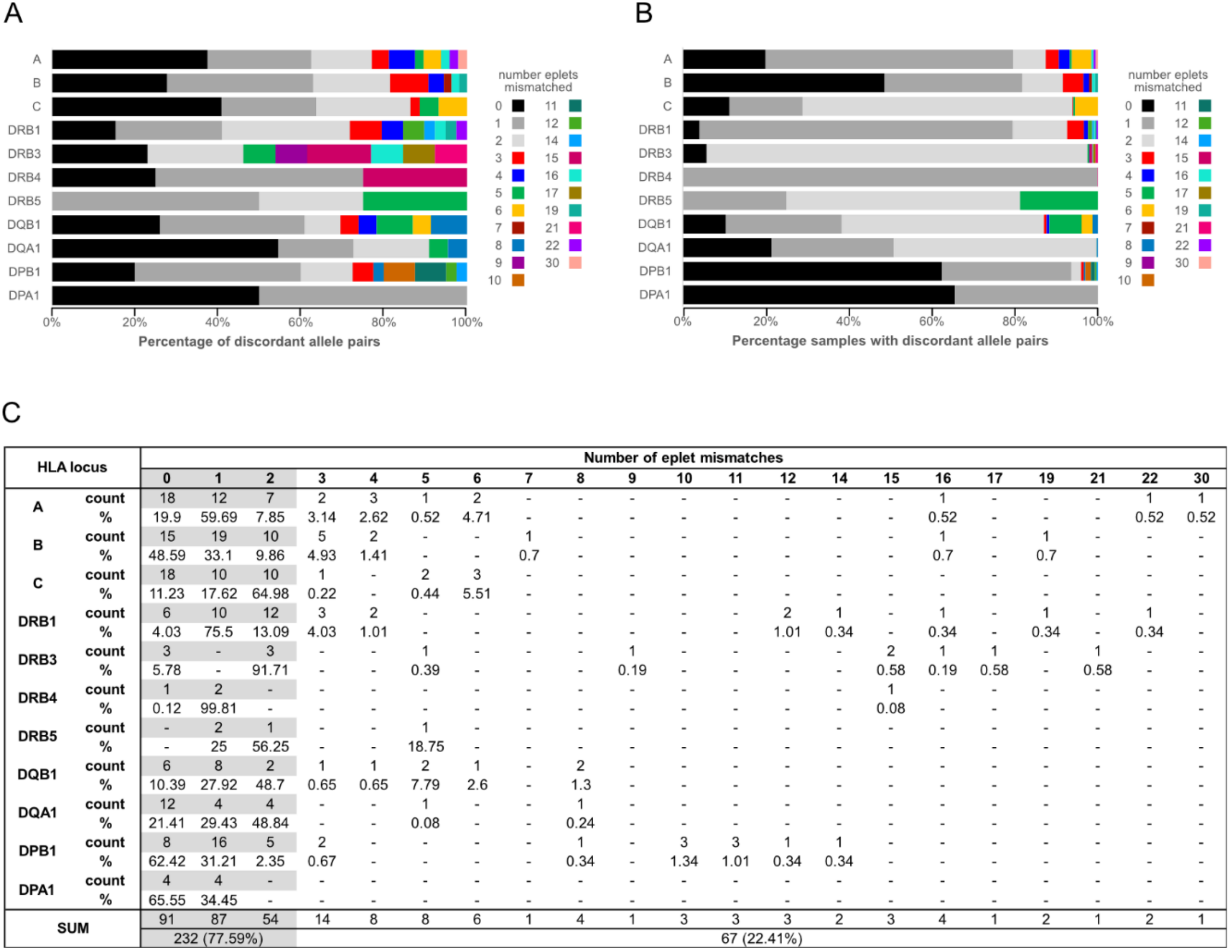
Eplet mismatch distributions. **(A)** eplet mismatch distribution based on unique rSSO–NGS allele-pair types, where each distinct combination is counted once. Gray tones correspond to 0–2 mismatches, which account for the majority of observed discrepancies, particularly at Class I loci [HLA-**(A-C)**]. Distinct colors represent mismatch categories ≥3 eplets, highlighting less common but structurally meaningful discordances. **(B)** sample-weighted eplet mismatch distribution. Coloring as in panel **(A, C)** Table showing the values used for **(A, B)** Null or negative or questionable or novel alleles were excluded from the eplet mismatch calculation.

We assessed molecular mismatch by determining the number and frequency of eplet mismatches for each unique combination of discordant NGS type and the first allele of the rSSO-reported allele string (**Supplementary Table 12**). Eplets are shared amino acid motifs that correlate with antibody reactivity and form all or part of the antibody epitope. When assessing the number of unique discordant pairs contributing to each level of eplet mismatch, our analysis showed that 77.59% had two or fewer eplets mismatched (**Figure 1C**). The lowest level of mismatch was observed for HLA-DPA1, where all mismatches were 1 or 0 (**Figure 1C**; **Supplementary Table 12**).

We also determined the percentage of samples with discordant pairs falling into each eplet mismatch level (**Figure 1B**). This indicated that high eplet mismatch pairs were generally found at very low frequencies, and the predominant mismatch frequencies were 2 eplets or fewer. DRB5 had the lowest frequency of samples with eplet mismatches of 2 or fewer at 81.25%. The other genes varied from 87% (DQB1) to 100% (DPA1). Second to HLA-DPA1 was HLA-DRB4, which, despite the large number of mismatched alleles (**Table 3**), had overall low levels of molecular mismatch, with all but one combination having one or zero eplet mismatches (**Figure 1A**; **Supplementary Table 12**). Those combinations accounted for 99.93% of the discordant alleles. The exception was the 15 eplet mismatch found for two alleles, where NGS typing found HLA-DRB4*01:05, and the first allele of the rSSO-reported allele string was HLA-DRB4*01:01. The discordant allele was present in the rSSO string in both instances.

**Table 3:** Samples categorized by concordance and eplet mismatch.

HLA locus	Concordant	2 eplet mismatches or fewer	>2 eplet mismatches	Undefined*
	% (count)	% (count)	% (count)	% (count)
A	97.9 (9281)	1.76 (167)	0.25 (24)	0.04 (4)
B	98.5 (9334)	1.37 (130)	0.13 (12)	0 (0)
C	95.2 (9020)	4.5 (426)	0.3 (28)	0.02 (2)
DRB1	96.8 (9177)	2.91 (276)	0.23 (22)	0.01 (1)
DRB3	94.5 (8954)	5.34 (506)	0.14 (13)	0.03 (3)
DRB4	72.0 (6827)	27.18 (2576)	0.02 (2)	0.75 (71)
DRB5	99.6 (9442)	0.14 (13)	0.03 (3)	0.19 (18)
DQB1	98.3 (9318)	1.41 (134)	0.21 (20)	0.04 (4)
DQA1	86.7 (8220)	13.12 (1243)	0.04 (4)	0.1 (9)
DPB1	96.8 (9173)	3.02 (286)	0.13 (12)	0.05 (5)
DPA1	98.7 (9350)	1.26 (119)	0 (0)	0.07 (7)

*Undefined includes mismatches involving null or new alleles not present in the eplet dataset.

The largest outlier, by percentage of discordant alleles greater than two eplets, was HLA-DRB5, with a 5 eplet mismatch representing 18.75% of the discordant DRB5 alleles. However, despite its seemingly high frequency, this represents only 3 of the 9,476 total alleles. In fact, given the high levels of concordance, the samples with eplet mismatches greater than 2 are less than 0.3% of the total alleles for each of the 11 genes analyzed. The highest frequency (0.3%) was observed for HLA-C, primarily due to three distinct 6 eplet mismatches (**Table 3**, **Figure 1A**).

## Discussion

Assessment of molecular mismatch between donor and recipient HLA alleles is an important and promising tool for personalized risk determination in transplantation. However, the time constraints imposed by the need for rapid organ allocation in deceased donor transplants generally limit HLA typing to low- or intermediate-resolution methods, thereby affecting the utility of molecular mismatch analysis. Imputation tools have been developed, notably HLAMatchmaker (6) and HaploStats (https://www.haplostats.org), which use haplotype-frequency and linkage disequilibrium data to generate allele-level haplotype predictions. These tools have primarily been used with low-resolution input data, and their output has shown limited accuracy, particularly in non-Caucasian populations.

Cohen et al. used existing patient/donor data to benchmark the use of imputation in molecular mismatch analysis (21). Their study constructed a dataset of surrogate donor-recipient pairs and assessed changes in molecular mismatch between high-resolution typing and imputed low-resolution results. Their conclusion was that, despite the prediction inaccuracies reported by Engen et al. (15) and Senev et al. (22), the use of imputation did not materially affect the classification of donor–recipient pairs into broad molecular mismatch categories. In this study, we used a different approach to examine whether using the first allele of the MAC-coded ambiguity string generated from rSSO typing was a sufficient proxy for high-resolution typing by assessing the concordance between NGS and rSSO results obtained from the same sample. This result was then used to assess the degree of molecular mismatch introduced in discordant results.

HLA alleles are named with four fields, each one representing an increase in resolution considering the protein produced by the gene (https://hla.alleles.org/pages/nomenclature/naming_alleles/). The highest-resolution, four-field, provides a complete gene sequence and can identify not only variations in the protein sequence encoded by the gene, but also additional variations that can alter protein expression. **Table 4** provides a summary comparison of low-, intermediate-, and high-resolution typing methods, including the time required and additional data needed to maximize output accuracy. An in-depth review of methods has been recently published (23). Current clinical practice worldwide generally provides intermediate-resolution typing, as the reagents and equipment are readily available and cost-effective. High-resolution methods are becoming more widely available, but there are still barriers of equipment cost and reagent availability that limit their broad adoption. Archival records can be a mix of resolutions, depending on the time and capabilities available at the time of assay.

**Table 4:** Comparison of HLA typing methods.

Resolution	LOW	INTERMEDIATE	HIGH
Technique	serology	rSSO, SSP, SBT	NGS
Input	viable cells	DNA	DNA
Access	broad	broad	limited
Time	rapid	rapid	slower
Level	one-field	two-field	four-field
Ambiguity	group only	reported with MAC codes	none
New alleles	no	limited	yes
Null alleles	lack of expression	no	yes
Match accuracy increase	imputation using population reference	this study	none needed
Additional data required	ethnicity	none	none
Effect on molecular mismatch	limited	limited	none

Our findings demonstrate that the first allele listed in the rSSO-derived multiple allele code (MAC) string provides a reliable surrogate for high-resolution HLA typing when estimating molecular mismatch. Although perfect allele-level concordance with next-generation sequencing (NGS) was not observed, discordant calls were almost uniformly associated with low eplet mismatch burdens. Prior studies have consistently shown that low eplet mismatch levels are associated with reduced alloimmune risk, supporting the clinical relevance of this approach (10, 13, 14).

Large clinical cohorts have established that molecular mismatch thresholds—rather than exact allele identity—are the primary determinants of alloimmune outcomes. Investigations by Wiebe et al. and Senev et al. (10, 13, 14) demonstrated that the risks of *de novo* donor-specific antibody formation and antibody-mediated rejection increase substantially only beyond defined eplet mismatch cutoffs. In our analysis, although discordant allele pairs exhibited a wide theoretical range of eplet mismatches (0–30), the majority (77.59%) involved two or fewer mismatched eplets. Across individual loci, the proportion of discordant pairs with two or fewer eplet mismatches ranged from 81.3% for HLA-DRB5 to 100% for HLA-DPA1, with most loci exceeding 90%. When the complete dataset was considered, allele pairs with more than two mismatched eplets accounted for less than 0.3% of all comparisons, underscoring the rarity of potentially high-risk discordance. Within this framework, nearly all discordant cases identified in this study would be classified as low risk for alloimmune injury.

Allele discordance was due to several factors. A major contributing factor is the incomplete resolution provided by the rSSO assay. This assay system relies on a predefined set of SSOs. These probe sets do not completely cover each gene, as their design has focused on common sites of variation and therefore cannot discriminate alleles that differ at sites outside the regions covered by the SSO. For example, the DRB4*01:03N allele is not discriminated from the DRB4*01:03 allele, as the variation causing lack of expression is not part of the SSO set and was always classed as discordant and excluded from the allele string in this study. The SSO probes are concentrated in exons 2 and 3 of the class I genes and exon 2 of the class II genes, so much of the variation outside of those regions is missed. Another source of ambiguity arises because the phasing of variation is not available for SSO, and the possible ambiguous allele combinations are then included in the final MAC codes. These variations are dependent on the specific SSO probe set used. In our dataset, two of the largest groups of discordant alleles were found for DRB4 and DQA1. In both cases, the first allele of the MAC code was the 01:01 allele, and the NGS typed allele(s) were higher on the list. For DQA1, it was a mix of the 01:03, 01:04, and 01:05 alleles, all present at reasonable frequency in some worldwide populations (https://www.allelefrequencies.net (24)) whereas for DRB4, it was the presence of the 01:03 or 01:03N allele in almost all discordant samples. The DRB4*01:01 and DRB4*01:03 alleles differ by a cluster of substitutions resulting in three amino acid differences. This cluster of substitutions is found in a number of other alleles of both the same and different first field types, and the lack of phasing discrimination of these alleles makes it impossible.

The lack of racial or ethnic differences in allele concordance underscores the robustness and population-agnostic nature of this approach, addressing a key limitation of methods that depend on population-specific reference data. Unlike haplotype-based imputation, rSSO provides a laboratory-based strategy for allele selection that operates independently of population reference panels and linkage disequilibrium assumptions. Because allele selection is derived directly from experimental measurements, this approach minimizes biases that can compromise accuracy in ancestrally diverse or underrepresented populations, where imputation methods may perform unevenly. By leveraging data already generated through routine rSSO typing, this strategy offers a computationally straightforward and immediately deployable means of estimating molecular mismatch without the need for additional inference or sequencing.

Recent advances in long-read sequencing, particularly the application of Oxford Nanopore Technologies platforms, have significantly expanded the feasibility of high-resolution HLA typing in the deceased donor organ transplant setting (25). Nanopore sequencing enables real-time generation of long reads spanning entire HLA genes, allowing unambiguous phase resolution across highly polymorphic regions that are difficult to resolve using short-read technologies (26). This capability supports rapid assignment of HLA alleles beyond two-field resolution directly from donor DNA, even within time-sensitive organ procurement workflows. Importantly, the portability, scalability, and reduced infrastructure requirements of Nanopore systems make them well-suited for deployment in organ procurement organizations and transplant laboratories. As turnaround times continue to improve and analytical pipelines mature, Nanopore-based HLA typing has the potential to enable near–real-time, high-resolution donor typing, facilitating more precise molecular mismatch assessment, improving donor–recipient matching, and advancing precision medicine in deceased donor allocation.

There were some limitations to our study approach. We compared typing results from individual subjects using NGS as the true result, then determined molecular mismatches in a unidirectional manner. This was done to simulate situations with known recipient and allele information from donor or archival data available at intermediate resolution. As this was a single-center study, the intermediate typing data were consistent with this center’s practice, and inclusion of data from other centers, particularly those using different rSSO vendors, could result in a slightly different composition of the allelic string. Although the study cohort was racially and ethnically diverse and showed no differences in allele concordance across groups, it may not fully capture all ancestries present in broader transplant populations. As such, generalizability to populations that remain underrepresented in this dataset cannot be assumed and warrants further validation in independent and more globally representative cohorts.

Collectively, our results support the use of the first allele in the rSSO MAC string as a practical proxy for high-resolution HLA typing in molecular mismatch assessment when NGS data are unavailable. This approach has important implications for both clinical practice and research. In time-sensitive settings such as deceased donor allocation, it enables real-time molecular mismatch estimation without delaying organ placement. In retrospective studies, it facilitates the inclusion of large historical cohorts that lack sequencing-based HLA data, thereby expanding opportunities to evaluate molecular mismatch as a biomarker for transplant outcomes.

## Data Availability

The original contributions presented in the study are included in the article/**Supplementary Material**. Further inquiries can be directed to the corresponding author.
